# Racial disparities negatively impact outcomes in early‐onset colorectal cancer independent of socioeconomic status

**DOI:** 10.1002/cam4.4276

**Published:** 2021-10-14

**Authors:** Suneel D. Kamath, Nataly Torrejon, Wei Wei, Katherine Tullio, Kanika G. Nair, David Liska, Smitha S. Krishnamurthi, Alok A. Khorana

**Affiliations:** ^1^ Cleveland Clinic Lerner College of Medicine Cleveland Clinic Taussig Cancer Institute Cleveland Ohio USA; ^2^ Department of Internal Medicine Cleveland Clinic Foundation Cleveland Ohio USA; ^3^ Department of Quantitative Health Sciences Cleveland Clinic Cleveland Ohio USA

**Keywords:** disparities, early‐onset colorectal cancer, health disparities, racial disparities, social determinants of health

## Abstract

**Background:**

The incidence of colorectal cancer (CRC) in patients under age 50 is rising for unclear reasons. We examined the effects of socioeconomic factors on outcomes for patients with early‐onset CRC compared to late‐onset CRC.

**Methods:**

Patients with CRC from 2004 to 2015 in the National Cancer Database were included and categorized by age (under or over 50 years). Differences in demographic and socioeconomic factors, disease characteristics, and survival outcomes between early‐onset versus late‐onset CRC patients were assessed by Chi‐squared test and Cox models.

**Results:**

The study population included 1,061,204 patients, 108,058 (10.2%) of whom were under age 50. The proportion of patients diagnosed under age 50 increased over time: 9.4% in 2004–2006, 10.1% in 2007–2009, 10.5% in 2010–2012, and 10.7% in 2013–2015 (*p *< 0.0001). Early‐onset CRC patients were more likely to be Black (15.1% vs. 11.3%) or Hispanic (8.6% vs. 4.6%) and to present with stage 4 disease (24.9% vs. 17.0%), *p *< 0.0001 for all. Black patients had the worst median OS (58.3 months) compared to White (67.0 months), Hispanic (91.6 months), or Asian (104.9 months) patients, *p* < 0.0001. Within the subgroup of early‐onset CRC patients with private insurance, Black patients had worse OS compared to White patients, even in communities with higher income and education status.

**Conclusions:**

Early‐onset CRC continues to increase. Patients with early‐onset CRC are more likely to be Black or Hispanic and to present with stage 4 cancer. Early‐onset Black patients showed worse OS compared to White patients in all income subgroups, even with private insurance.

## Graphical abstract

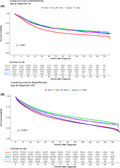

**Lay Summary**

The number of people under age 50 that are diagnosed with colorectal cancer continues to increase for unknown reasons. Patients with early‐onset colorectal cancer are more likely to be Black or Hispanic and to present with stage 4 cancer. Black patients with early‐onset colorectal cancer have worse outcomes and shorter survival compared to White patients, even with private insurance and in communities with higher income and education status.

## 1 INTRODUCTION

Colorectal cancer (CRC) is the third most common cancer and second leading cause of cancer death in the United States.^1^, ^2^ While incidence and mortality from CRC are declining in older patients, the incidence of early‐onset CRC (eoCRC) in patients under age 50 has increased by 1.3% annually over the last two decades.^1^, ^2^, ^3^, ^4^ Currently, eoCRC represents more than 10% of CRC diagnoses, and incidence rates for colon and rectal cancer are projected to rise by 90% and 124%, respectively, by 2030.^2^, ^3^, ^4^, ^5^, ^6^ The alarming rise of eoCRC was a significant contributor to the 2018 update in American Cancer Society CRC screening guidelines, which now recommend initiating CRC screening at age 45.^7^ The US Preventive Services Task Force also released a draft statement indicating strong consideration for lowering their recommended age to initiate CRC screening to age 45.

Early‐onset CRC has notable differences in epidemiology, tumor characteristics, and prognosis from late‐onset CRC (loCRC). Early‐onset CRC appears to affect non‐White patients at higher rates compared to White patients.^6^ Early‐onset CRCs are more likely to present with advanced or metastatic disease, with left‐sided tumors and with aggressive histologies such as mucinous or signet ring cells.^6^, ^8^, ^9^, ^10^ From a molecular perspective, eoCRCs appears to have lower rates of sporadic BRAF, APC, and NRAS mutations while having higher rates of genomic alterations associated with hereditary syndromes such as microsatellite instability (MSI).^11^, ^12^ While eoCRC patients are more likely to be treated with more aggressive interventions, these interventions translate to only modest improvements in outcomes.^9^

The cause of the rise in incidence of eoCRC remains unknown, but likely many factors from environmental exposures and tumor biology differences play a role. Early‐onset CRC patients are more likely to be Black or Hispanic, two groups that experience socioeconomic barriers and health disparities in cancer care.^13^ However, less is known about how race, ethnicity, and socioeconomic factors specifically impact outcomes in the eoCRC population. In this study, we examined data from the National Cancer Database (NCDB) to measure differences in epidemiological trends and clinical outcomes between eoCRC and loCRC as well as to assess for socioeconomic differences between the groups.

## 2 METHODS

The study cohort was obtained from the NCDB, a joint program of the Commission on Cancer (CoC) of the American College of Surgeons and the American Cancer Society. The NCDB is a hospital‐based, prospectively collected outcomes database comprising approximately 70% of all new invasive cancer diagnoses and more than 1500 CoC‐accredited facilities in the United States. Data collection is standardized based on the Facility Oncology Registry Data Standards (FORDS) and data were generated using the Participant Use File program. For this study, adult patients diagnosed with CRC from 2004 to 2015 were included. As the dataset is de‐identified and publically available, this study was granted exempt status by the institutional review board of the Cleveland Clinic Taussig Cancer Institute.

Data abstracted included age, sex, race, ethnicity, community median income, insurance status, community education level, county urbanization, year of diagnosis, American Joint Committee on Cancer (AJCC) stage, tumor grade, cancer center type, and treatment modalities used. Patient self‐reported race is defined and categorized in the NCDB as non‐Hispanic White, Black, Asian, and Other/unknown. The NCDB categorizes patient self‐reported ethnicity as either of Hispanic origin or not. For our analyses, race and ethnicity were evaluated as one variable. The NCDB measures community education level by the percentage of people in a community that have not completed at least a high school degree, which is categorized as <7.0%, 7.0%–12.9%, 13.0%–20.9%, and >21.0%. Level of comorbidity was measured using the Charlson–Deyo score. Patients were then categorized as eoCRC (age <50 years) and loCRC (age ≥50 years) based on age at CRC diagnosis.

Statistical analyses evaluated associations between patient, socioeconomic, and disease attributes with overall survival. Baseline characteristics were summarized using percentages for categorical variables and medians for continuous variables. Overall survival was estimated using the Kaplan–Meier method. Baseline characteristics between eoCRC and loCRC patients were compared using the Chi‐squared test for categorical variables and the Wilcoxon rank sum test for continuous variables. The Cox proportional hazards model was used for univariate and multivariate regression analyses of overall survival, which included all baseline characteristics and age <50 years versus age ≥50 years at diagnosis as variables. There were significant interaction effects on OS between race/ethnicity and income, insurance status, and education, therefore, multivariate Cox modeling was performed to assess the effect of race/ethnicity on overall survival within both age groups after subgrouping by community median income, insurance status, and community education level. All tests were two‐sided and subgroup analyses used Bonferroni correction to control overall type I error rate at 5%. Data analyses were performed using SAS Studio 3.7 (SAS Institute, Inc) and R version 4 (R Foundation).

## 3 RESULTS

The study population was comprised of 1,061,204 patients, 108,058 (10.2%) of whom had eoCRC. The median age at diagnosis for all patients was 68 years. In the total study population, 48.9% of patients were female and 51.1% were male. In terms of race, 79.1% of patients were White, 11.6% of patients were Black, and 2.7% of patients were Asian. In terms of ethnicity, 5.0% of patients self‐reported as Hispanic. The majority of patients had government insurance (60.5%) and lived in a metropolitan area (82.2%). Government insurance included all forms of Medicaid and Medicare. In terms of stage, 68.4% of patients presented with stages I–III cancer, 17.8% had stage IV cancer, and 13.9% had an unknown stage. These and other baseline characteristics are summarized in Table 1.

**TABLE 1:** Baseline characteristics of early‐onset and late‐onset CRC patients

	Overall, *n* (%)	Age <50 years, *n* (%)	Age ≥50 years, *n* (%)
Total cases	1,061,204 (100%)	108,058 (10.2%)	953,146 (89.8%)
Sex^a^			
Female	519,091 (48.9%)	50,992 (47.2%)	468,099 (49.1%)
Male	542,113 (51.1%)	57,066 (52.8%)	485,047 (50.9%)
Race/ethnicity^b^			
White	839,284 (79.1%)	75,903 (70.2%)	763,381 (80.1%)
Black	123,531 (11.6%)	16,280 (15.1%)	107,251 (11.3%)
Hispanic	52,951 (5.0%)	9335 (8.6%)	43,616 (4.6%)
Asian	28,884 (2.7%)	4271 (4.0%)	24,613 (2.6%)
Other	16,554 (1.6%)	2269 (2.1%)	14,285 (1.5%)
Median annual income^a^			
<$63,000	731,342 (68.9%)	72,232 (66.8%)	659,110 (69.1%)
≥$63,000	329,862 (31.1%)	35,826 (33.2%)	294,036 (30.9%)
Education^a^			
≥21% with no HS degree	240,143 (22.6%)	26,589 (24.6%)	213,554 (22.4%)
<21% with no HS degree	821,061 (77.4%)	81,469 (75.4%)	739,592 (77.6%)
Insurance^b^			
Private	364,992 (34.4%)	76,837 (71.1%)	288,155 (30.2%)
Government	642,483 (60.5%)	19,570 (18.1%)	622,913 (65.4%)
Insurance^b^			
Uninsured	34,807 (3.3%)	8920 (8.3%)	25,887 (2.7%)
Unknown	18,922 (1.8%)	2731 (2.5%)	16,191 (1.7%)
Residence area^a^			
Metro	872,977 (82.3%)	90,233 (83.5%)	782,744 (82.1%)
Urban	141,263 (13.3%)	13,299 (12.3%)	127,964 (13.4%)
Rural	20,242 (1.9%)	1634 (1.5%)	18,608 (2.0%)
Unknown	26,722 (2.5%)	2892 (2.7%)	23,830 (2.5%)
Facility type^a^			
Academic	290,752 (27.4%)	29,297 (27.1%)	261,455 (27.4%)
Community	770,452 (72.6%)	78,761 (72.9%)	691,691 (72.6%)
Charlson–Deyo score^b^			
0	746,247 (70.3%)	95,404 (88.3%)	650,843 (68.3%)
≥1	314,957 (29.7%)	12,654 (11.7%)	302,303 (31.7%)
AJCC stage at diagnosis^b^			
Stage I–III	725,387 (68.4%)	69,833 (64.6%)	655,554 (68.8%)
Stage IV	188,535 (17.8%)	26,891 (24.9%)	161,644 (17.0%)
Unknown	147,282 (13.9%)	11,334 (10.5%)	135,948 (14.3%)
Tumor grade^a^			
Grade 1–2	705,026 (66.4%)	70,958 (65.7%)	634,068 (66.5%)
Grade 3	181,252 (17.1%)	19,872 (18.4%)	161,380 (16.9%)
Unknown	174,926 (16.5%)	17,228 (15.9%)	157,698 (16.5%)

Abbreviations: HS, high school; n, number.

^a^ indicates *p *< 0.0001, but not considered clinically meaningful.

^b^ indicates *p *< 0.0001 and clinically meaningful.

The proportion of patients diagnosed with eoCRC increased over time: 9.4% from 2004 to 2006, 10.1% from 2007 to 2009, 10.5% from 2010 to 2012, and 10.7% from 2013 to 2015 (*p *< 0.0001). Compared to loCRC patients, a higher proportion of eoCRC patients were Black (15.1% vs. 11.3%), Hispanic (8.6% vs. 4.6%), or Asian (4.0% vs. 2.6%) while a smaller proportion were White (70.2% vs. 80.1%), *p *< 0.0001 for all. A higher proportion of eoCRC patients were diagnosed with stage IV CRC compared to loCRC patients (24.9% vs. 17.0%, *p *< 0.0001). The majority of eoCRC patients had private insurance (71.1%) while the majority of loCRC patients had government insurance (65.4%), *p *< 0.0001. The eoCRC patients were also more likely to be uninsured compared to loCRC patients (8.3% vs. 2.7%, *p *< 0.0001). These data and comparisons of other socioeconomic and cancer‐specific factors are shown in Table 1. An equal proportion of the eoCRC and loCRC patients underwent surgery as part of their treatment (85.7% vs. 84.9%). A larger proportion of eoCRC patients were treated with radiation or chemotherapy compared to loCRC patients (23.6% vs. 12.9% and 65.8% vs. 36.7%, respectively, *p *< 0.0001 for both comparisons).

**FIGURE 1:**
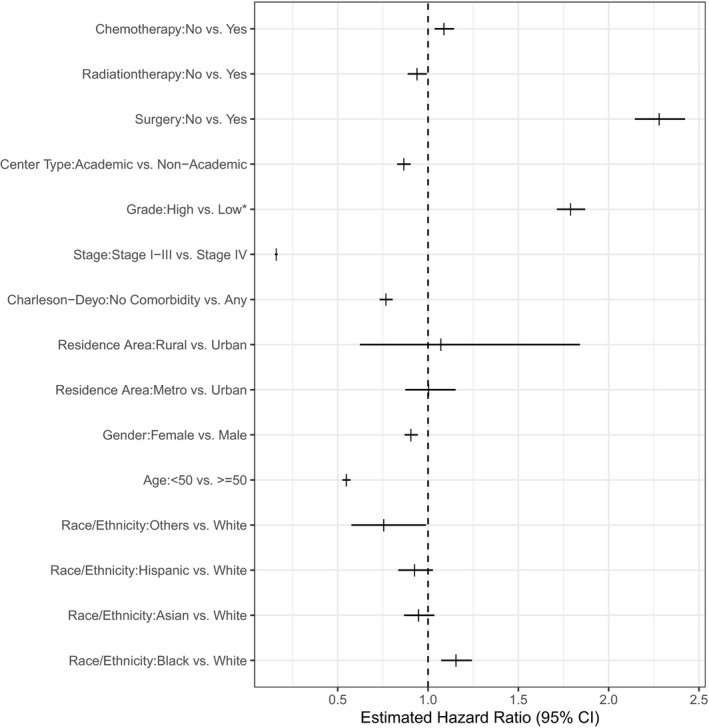
Forest plot demonstrating associations between patient, socioeconomic, and disease attributes with overall survival

Compared to loCRC patients, eoCRC patients had significantly longer median overall survival (157.4 vs. 64.2 months, *p *< 0.0001). As expected, earlier stage at diagnosis, lower tumor grade, younger age, lower Charlson–Deyo comorbidity score, private insurance status, receiving care at an academic center, higher income, and higher community education level were all associated with improved overall survival. These data and others are summarized in Figure 1.

In the total study population, Black patients experienced worse median overall survival compared to White patients (58.3 vs. 67.0 months, *p *< 0.0001). Conversely, patients of Asian, Hispanic, or Other origins had better overall survival compared to White patients (104.9, 91.6 and 89.4 months, respectively, *p* < 0.0001 for all). The interactions between race/ethnicity and treatment status were not statistically significant, indicating the effects of treatment on OS were the same across racial and ethnic groups.

**FIGURE 2:**
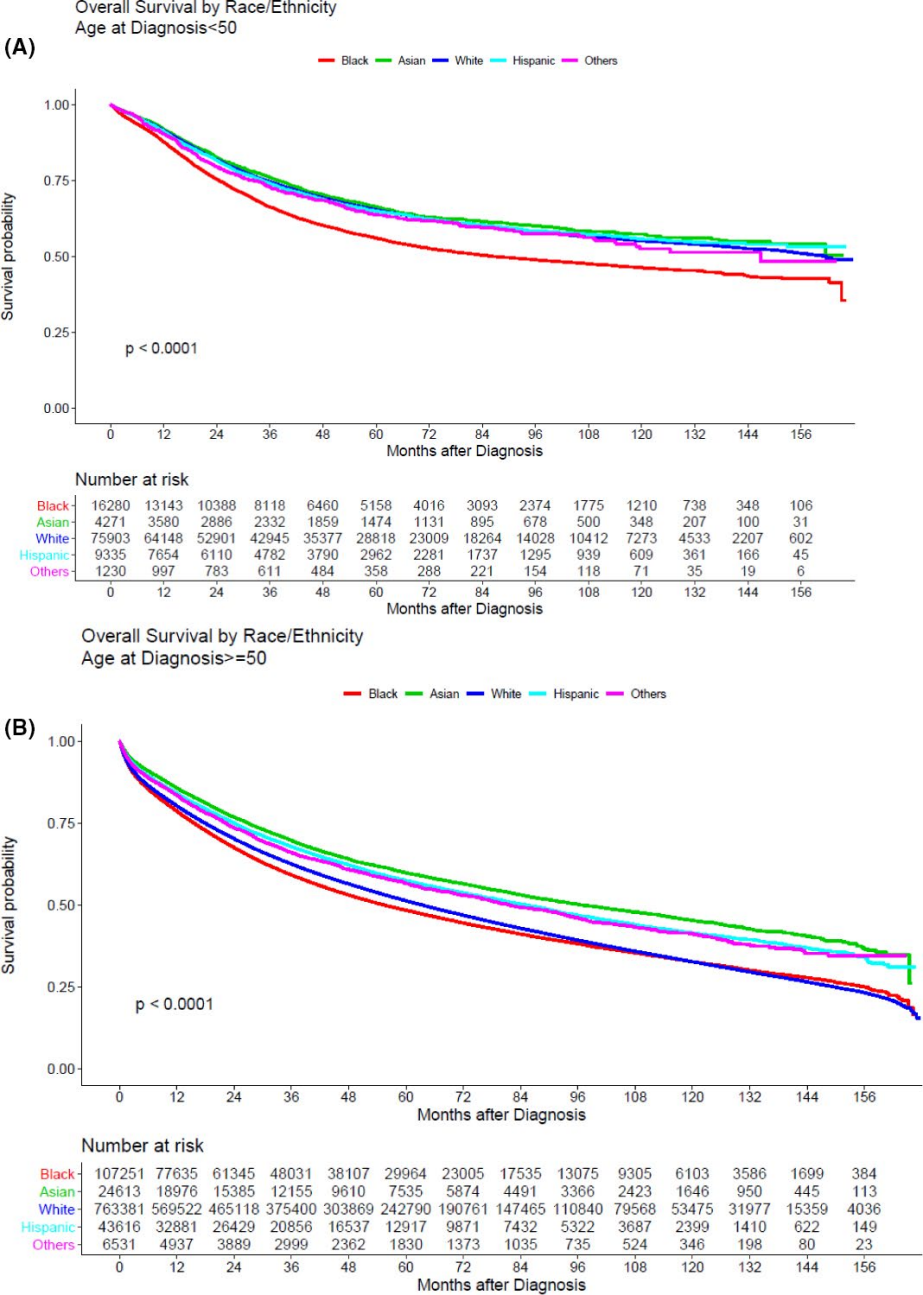
Overall survival by race/ethnicity and stratified by age <50 (A) versus age ≥50 years (B)

The disparities in survival outcomes were most pronounced among the eoCRC population. Specifically, Black patients experienced significantly worse overall survival compared to patients of White, Asian, Hispanic, and Other races/ethnicities. In the loCRC population, Black and White patients experienced similar outcomes, while patients of Asian, Hispanic, and Other races/ethnicities had better outcomes. These data are illustrated in Figure 2.

Within the subgroup of eoCRC patients with private insurance, multivariable Cox models showed Black patients had worse OS compared to White patients at multiple community income and education levels, after adjusting for gender, residence area, comorbidity, stage, tumor grade, treatment facility type, and treatments given. In communities with median income <$38,000 and more than 21% of patients without a high school degree, Black patients had worse overall survival compared to White patients (OS HR: 1.31, 95% CI: 1.18–1.47, *p *< 0.0001). Black patients experienced worse overall survival compared to White patients in communities with median income between $38,000–$63,000 and 7.0%–12.9% of patients without a high school degree (OS HR: 1.41, p5% CI: 1.25–1.59, *p *< 0.0001). In communities with median income >$63,000 (highest income bracket) and 7.0%–12.9% of patients without a high school degree, Black patients still experienced worse overall survival compared to White patients (OS HR: 1.38, 95% CI: 1.20–1.60, *p *< 0.0001). In communities with median income >$63,000 (highest income bracket) and <7.0% of patients without a high school degree (highest educational level), the disparity in survival for Black patients was mitigated and did not reach statistical significance (HR: 1.16, 95% CI: 1.0–1.34, *p* = 0.05). These data and other statistically significant multivariable Cox models comparing different community income and education levels by race or ethnicity are shown in Table 2. Additional models are shown in Table S1.

**TABLE 2:** Comparison of outcomes by race in early‐onset CRC patients with private insurance, stratified by community income and education level

Community median income	Community proportion without high school degree	Comparison	OS HR (95% CI)	*p* value
<$38,000	≥21%	Black versus White Hispanic versus White Asian versus White	1.31 (1.18–1.47) 0.89 (0.75–1.06) 1.45 (0.98–2.13)	<0.0001 0.18 0.06
<$38,000	13.0%–20.9%	Black versus White Hispanic versus White Asian versus White	1.38 (1.18–1.62) 1.02 (0.65–1.60) 1.80 (0.93–3.51)	<0.0001 0.93 0.08
$38,000–63,000	13.0%–20.9%	Black versus White Hispanic versus White Asian versus White	1.31 (1.19–1.44) 0.92 (0.78–1.08) 1.31 (1.04–1.65)	<0.0001 0.32 0.02*
$38,000–63,000	7.0%–12.9%	Black versus White Hispanic versus White Asian versus White	1.41 (1.25–1.59) 0.81 (0.64–1.02) 1.05 (0.82–1.35)	<0.0001 0.07 0.70
>$63,000	7.0%–12.9%	Black versus White Hispanic versus White Asian versus White	1.38 (1.20–1.60) 0.89 (0.72–1.09) 1.00 (0.84–1.20)	<0.0001 0.25 0.95
>$63,000	<7.0%	Black versus White Hispanic versus White Asian versus White	1.16 (1.0–1.34) 0.86 (0.69–1.06) 0.97 (0.84–1.11)	0.05* 0.16 0.64

Abbreviations: HR, hazard ratio; OS, overall survival.

* Not statistically significant as only *p* ≤ 0.0002 was considered statistically significant after Bonferroni correction.

Black patients with eoCRC who had government insurance or were uninsured experienced similar outcomes to White patients with the same insurance status regardless of community income or education level. Black patients with loCRC did not experience disparities in overall survival compared to White patients at comparable strata of community income, education, and insurance status. Asian and Hispanic patients had equivalent or better OS compared to White patients at comparable strata of community income, education, and insurance status, regardless of age at diagnosis.

## 4 DISCUSSION

In a large, national, cancer outcomes database, we showed that the rise of eoCRC disproportionally affects non‐White patients and Black patients experience worse outcomes, including those living in higher income and more educated communities. Our findings are somewhat discordant from other studies that have shown the greatest increase in eoCRC incidence in White patients.^14^, ^15^ However, it is important to note that these studies reported on differences in the rate of rise between racial/ethnic groups as opposed to the proportion of eoCRC cases accounted for by each racial/ethnic group. Additionally, many of these studies used the Surveillance, Epidemiology, and End Results program data, which includes a different, population‐based dataset with fewer patients compared to NCDB.^16^

The causes of the rising incidence of eoCRC remain incompletely understood. There are likely a number of factors, including higher rates of obesity, increased antibiotic use, altered gut microbiome, increase consumption of red/processed meats, and high‐fructose corn syrup and physical inactivity.^3^, ^17^, ^18^ Gut microbial dysbiosis caused by antibiotic use early in life or a diet high in red meat and low in fiber has been associated with increased risk for colorectal adenomas and cancer in younger patients.^19^, ^20^ As the NCDB does not include diet or medication histories, it is possible that racial or ethnic differences in diet and antibiotic use could account for part of the increased risk for colorectal cancer in non‐White patients. The known risk factors for eoCRC explain part of the rising incidence of eoCRC, but there are likely many others that need to be elucidated.

This study confirms prior research showing Black patients with CRC experience worse outcomes, but interestingly, it demonstrates disparities in outcomes even for Black patients with private insurance living in communities with higher income and education status.^21^ Health insurance status, a surrogate for access to medical care, is an important social determinant of health. Many prior studies have shown that private insurance status is associated with improved cancer detection, more guideline‐concordant care, and better outcomes, while having Medicaid or no insurance was associated with worse outcomes.^22^, ^23^, ^24^, ^25^ In this analysis, patients with private insurance survived more than twice as long as those with government insurance or without insurance. However, our data show that Black patients with eoCRC may not benefit equally from the improved access to care associated with private insurance status compared to other patients.

These results should not be interpreted as diminishing the value of efforts to improve access to care for Black patients. Our results showed that Black patients with eoCRC living in communities with both the highest median income and highest education status experienced comparable outcomes as socioeconomically similar White patients, suggesting that addressing poverty and access issues are still critically important. Prior research has shown that while tumor characteristics may explain 25% of the survival disparity, health insurance coverage differences account for nearly half of it.^24^ A deeper, more granular evaluation of access issues for young patients with private insurance may identify new barriers that can be addressed.

This study does not specifically address potential causes for the observed disparities, but there are many possible contributors. Private insurances vary significantly in their cost and coverage based on insurer and plan. Though the data are limited, it is possible that certain plans do offer coverage, but may lead to underinsurance for certain aspects of care compared to others. For example, a study of the Florida Cancer Data System outcomes database evaluating survival differences for patients with commercial fee‐for‐service (FFS) versus health maintenance organization (HMO) found that patients with HMO insurance were less likely to receive chemotherapy and had greater mortality compared to patients with FFS plans. The results were independent of stage at diagnosis, patient comorbidities, and treatment modalities used.^23^ Other studies comparing outcomes between FFS and HMO plans did not arrive at the same conclusions, though many of these studies were restricted to the Medicare population and looked at single metropolitan areas.^26^, ^27^, ^28^, ^29^, ^30^ Another study of lower cost plans in the Affordable Care Act marketplace showed nearly 15% of plans lacked in‐network physicians for at least one specialty, including oncology. For two plans that did include at least one in‐network oncologist, the nearest one could be more than 50 miles away for some patients.^31^ Underinsurance could lead to delayed presentation to medical care and delayed diagnostic testing, ultimately leading to worse outcomes for Black patients with eoCRC. As the NCDB does not include data on rates of age‐appropriate screening or health maintenance visits, this study cannot determine if differences in access to medical care contributed to the observed results.

This study also found that lower community education status was associated with worse outcomes, even in communities with the same median income. This is consistent with prior studies that have shown that less educated patients are less likely to be offered colorectal cancer screening or to receive chemotherapy as part of their treatment.^32^, ^33^ Reduced health literacy could also impact the ability to participate in care and willingness to pursue certain treatments.^34^

Unconscious bias among health professionals may inadvertently contribute to worse outcomes for Black patients. Past and present systemic racism embedded in the healthcare system leading to diminished trust among Black patients may also contribute to the reduced benefits of private insurance, higher income, and better education for young Black patients compared to young White patients.^35^ Differences in environmental exposures, diet, microbiome, and tumor genomics likely all contribute as well.^17^, ^18^, ^36^

This study has several limitations. First, the NCDB is hospital‐based and not population‐based, meaning potential bias in types of hospitals included could bias our findings. However, given that the NCDB includes a large number of hospitals accounting for approximately 70% of all new cancer diagnoses, it is likely these results can be generalized to the broader population. Second, NCDB data does not include specific comorbidities such as obesity, chemotherapy regimens, or tumor genomics, which could vary by race or ethnicity and lead to disparate outcomes. Third, this study included many subgroup analyses, which could skew results compared to the total population. However, the large size of the NCDB dataset ensures even subgroups contain a large number of patients. The extensive subgroup analyses also ensure that the conclusions drawn in this study are robust and that the observed differences in outcomes can reasonably be attributed to racial and ethnic differences. Finally, the NCDB data on income and education status are based on geographic location, which may not account for racial/ethnic differences in income or education within each community.

In conclusion, the rising incidence of eoCRC disproportionately affects Black, Hispanic, and Asian patients. Black patients experience worse outcomes at any age, though the disparity is most pronounced in the early‐onset population. Alarmingly, Black patients with eoCRC showed worse OS compared to White patients in all income subgroups, even with private insurance. More work is needed to better understand and address these health disparities, particularly for younger patients.

## CONFLICTS OF INTEREST

Suneel Kamath––consulting or advisory role: Exelixis, Tempus. Nataly Torrejon––No disclosures. Wei Wei––No disclosures. Katherine Tullio––No disclosures. Kanika G. Nair––No disclosures. David Liska––No disclosures. Smitha S. Krishnamurthi––Consulting or Advisory Role––Array BioPharma, Research Funding––Abbvie (Inst); Bristol‐Myers Squibb; Celgene (Inst). Alok A. Khorana––has been paid honoraria directly by Janssen, Halozyme, Pfizer, Bayer, Nektar, and Medscape, currently or during the past 2 years. Dr. Khorana has been paid for any consulting or advisory role by Janssen, Halozyme, Bayer, Pfizer, Pharmacyte Biotech, Pharmacyclics, and Seattle Genetics.

## ETHICAL APPROVAL

As the dataset is de‐identified and publically available, this study was granted exempt status by the institutional review board of the Cleveland Clinic Taussig Cancer Institute.

## PRECIS

Patients with early‐onset colorectal cancer are more likely to be Black or Hispanic and to present with stage 4 cancer.

Within the subgroup of early‐onset CRC patients with private insurance, Black patients had worse OS compared to White patients, even in communities with higher income and education status.

## Supporting information

Table S1

## Data Availability

The data that support the findings of this study are openly available in the National Cancer Database at https://www.facs.org/quality‐programs/cancer/ncdb
