# Supplementary material for: Racial disparities negatively impact outcomes in early‐onset colorectal cancer independent of socioeconomic status
Source: Cancer Med. 2021 Oct 14;10(21):7542–50. doi: 10.1002/cam4.4276 (PMC8559495; doi:10.1002/cam4.4276)
Supplement: Supplementary file 1 — Table S1 [file CAM4-10-7542-s001.docx]

Supplemental Table 1.

| **Age** | **Community Median Income** | **Community without HS Degree** | **Insurance** | **Comparison** | **Hazard Ratio for OS** | **LCL** | **UCL** | **P-value** |
| --- | --- | --- | --- | --- | --- | --- | --- | --- |
| <50 | <38 K | ≥21.0% | Government | Black vs. White | 1.074 | 0.94 | 1.228 | 0.2956 |
| <50 | <38 K | ≥21.0% | Government | Asian vs. White | 0.83 | 0.538 | 1.282 | 0.4005 |
| <50 | <38 K | ≥21.0% | Government | Hispanic vs. White | 0.796 | 0.665 | 0.953 | 0.0132 |
| <50 | <38 K | ≥21.0% | Government | Others vs. White | 0.853 | 0.591 | 1.231 | 0.3956 |
| <50 | <38 K | ≥21.0% | Not Insured | Black vs. White | 1.183 | 0.977 | 1.433 | 0.0856 |
| <50 | <38 K | ≥21.0% | Not Insured | Asian vs. White | 0.389 | 0.141 | 1.072 | 0.068 |
| <50 | <38 K | ≥21.0% | Not Insured | Hispanic vs. White | 0.815 | 0.64 | 1.037 | 0.0961 |
| <50 | <38 K | ≥21.0% | Not Insured | Others vs. White | 0.926 | 0.29 | 2.963 | 0.8971 |
| <50 | <38 K | ≥21.0% | Private | Black vs. White | 1.314 | 1.176 | 1.467 | <.0001 |
| <50 | <38 K | ≥21.0% | Private | Asian vs. White | 1.445 | 0.98 | 2.132 | 0.0632 |
| <50 | <38 K | ≥21.0% | Private | Hispanic vs. White | 0.889 | 0.749 | 1.055 | 0.1788 |
| <50 | <38 K | ≥21.0% | Private | Others vs. White | 0.602 | 0.359 | 1.008 | 0.0535 |
| <50 | <38 K | 13.0-20.9% | Government | Black vs. White | 1.069 | 0.883 | 1.294 | 0.4933 |
| <50 | <38 K | 13.0-20.9% | Government | Asian vs. White | 0.942 | 0.383 | 2.316 | 0.8969 |
| <50 | <38 K | 13.0-20.9% | Government | Hispanic vs. White | 0.934 | 0.589 | 1.483 | 0.7723 |
| <50 | <38 K | 13.0-20.9% | Government | Others vs. White | 1.034 | 0.523 | 2.043 | 0.924 |
| <50 | <38 K | 13.0-20.9% | Not Insured | Black vs. White | 0.993 | 0.722 | 1.366 | 0.9659 |
| <50 | <38 K | 13.0-20.9% | Not Insured | Asian vs. White | 2.231 | 0.617 | 8.065 | 0.221 |
| <50 | <38 K | 13.0-20.9% | Not Insured | Hispanic vs. White | 0.705 | 0.299 | 1.665 | 0.4257 |
| <50 | <38 K | 13.0-20.9% | Private | Black vs. White | 1.383 | 1.18 | 1.622 | <.0001 |
| <50 | <38 K | 13.0-20.9% | Private | Asian vs. White | 1.803 | 0.925 | 3.513 | 0.0833 |
| <50 | <38 K | 13.0-20.9% | Private | Hispanic vs. White | 1.02 | 0.649 | 1.601 | 0.9329 |
| <50 | <38 K | 13.0-20.9% | Private | Others vs. White | 0.829 | 0.41 | 1.678 | 0.6026 |
| <50 | <38 K | 7.0-12.9% | Government | Black vs. White | 1.431 | 0.696 | 2.939 | 0.3296 |
| <50 | <38 K | 7.0-12.9% | Government | Hispanic vs. White | 2.579 | 0.787 | 8.449 | 0.1175 |
| <50 | <38 K | 7.0-12.9% | Government | Others vs. White | 2.651 | 0.532 | 13.216 | 0.2342 |
| <50 | <38 K | 7.0-12.9% | Private | Others vs. White | 1.233 | 0.751 | 2.025 | 0.4075 |
| <50 | <38 K | 7.0-12.9% | Private | Asian vs. White | 1.382 | 0.182 | 10.503 | 0.7544 |
| <50 | <38 K | 7.0-12.9% | Private | Hispanic vs. White | 2.184 | 0.964 | 4.951 | 0.0613 |
| <50 | <38 K | 7.0-12.9% | Private | Others vs. White | 5.172 | 1.137 | 23.523 | 0.0335 |
| <50 | <38 K | <7.0% | Government | Black vs. White | 0.17 | 0.003 | 10.648 | 0.401 |
| <50 | <38 K | <7.0% | Private | Black vs. White | 5.726 | 0.669 | 49.023 | 0.1112 |
| <50 | 38-63 K | ≥21.0% | Government | Black vs. White | 1.131 | 0.938 | 1.364 | 0.1959 |
| <50 | 38-63 K | ≥21.0% | Government | Asian vs. White | 0.764 | 0.57 | 1.024 | 0.072 |
| <50 | 38-63 K | ≥21.0% | Government | Hispanic vs. White | 0.824 | 0.691 | 0.982 | 0.0307 |
| <50 | 38-63 K | ≥21.0% | Government | Others vs. White | 0.569 | 0.234 | 1.385 | 0.214 |
| <50 | 38-63 K | ≥21.0% | Not Insured | Black vs. White | 0.85 | 0.642 | 1.125 | 0.2559 |
| <50 | 38-63 K | ≥21.0% | Not Insured | Asian vs. White | 0.546 | 0.308 | 0.969 | 0.0387 |
| <50 | 38-63 K | ≥21.0% | Not Insured | Hispanic vs. White | 0.606 | 0.473 | 0.777 | <.0001 |
| <50 | 38-63 K | ≥21.0% | Not Insured | Others vs. White | 0.44 | 0.061 | 3.184 | 0.4161 |
| <50 | 38-63 K | ≥21.0% | Private | Black vs. White | 1.036 | 0.891 | 1.204 | 0.6456 |
| <50 | 38-63 K | ≥21.0% | Private | Asian vs. White | 0.938 | 0.74 | 1.187 | 0.5922 |
| <50 | 38-63 K | ≥21.0% | Private | Hispanic vs. White | 0.873 | 0.759 | 1.006 | 0.0598 |
| <50 | 38-63 K | ≥21.0% | Private | Others vs. White | 0.85 | 0.467 | 1.547 | 0.595 |
| <50 | 38-63 K | 13.0-20.9% | Government | Black vs. White | 0.895 | 0.769 | 1.042 | 0.153 |
| <50 | 38-63 K | 13.0-20.9% | Government | Asian vs. White | 1.143 | 0.817 | 1.599 | 0.4344 |
| <50 | 38-63 K | 13.0-20.9% | Government | Hispanic vs. White | 0.769 | 0.614 | 0.963 | 0.0221 |
| <50 | 38-63 K | 13.0-20.9% | Government | Others vs. White | 0.649 | 0.413 | 1.022 | 0.0621 |
| <50 | 38-63 K | 13.0-20.9% | Not Insured | Black vs. White | 0.963 | 0.77 | 1.203 | 0.7387 |
| <50 | 38-63 K | 13.0-20.9% | Not Insured | Asian vs. White | 0.867 | 0.459 | 1.637 | 0.6597 |
| <50 | 38-63 K | 13.0-20.9% | Not Insured | Hispanic vs. White | 0.718 | 0.542 | 0.953 | 0.0219 |
| <50 | 38-63 K | 13.0-20.9% | Private | Black vs. White | 1.307 | 1.189 | 1.435 | <.0001 |
| <50 | 38-63 K | 13.0-20.9% | Private | Asian vs. White | 1.311 | 1.044 | 1.645 | 0.0195 |
| <50 | 38-63 K | 13.0-20.9% | Private | Hispanic vs. White | 0.92 | 0.781 | 1.083 | 0.3155 |
| <50 | 38-63 K | 13.0-20.9% | Private | Others vs. White | 1.006 | 0.647 | 1.564 | 0.9805 |
| <50 | 38-63 K | 7.0-12.9% | Government | Black vs. White | 1.134 | 0.96 | 1.34 | 0.1376 |
| <50 | 38-63 K | 7.0-12.9% | Government | Asian vs. White | 0.507 | 0.297 | 0.864 | 0.0125 |
| <50 | 38-63 K | 7.0-12.9% | Government | Hispanic vs. White | 0.855 | 0.622 | 1.176 | 0.3362 |
| <50 | 38-63 K | 7.0-12.9% | Government | Others vs. White | 1.278 | 0.761 | 2.144 | 0.3537 |
| <50 | 38-63 K | 7.0-12.9% | Not Insured | Black vs. White | 1.592 | 1.205 | 2.104 | 0.0011 |
| <50 | 38-63 K | 7.0-12.9% | Not Insured | Asian vs. White | 0.892 | 0.386 | 2.059 | 0.7885 |
| <50 | 38-63 K | 7.0-12.9% | Not Insured | Hispanic vs. White | 0.815 | 0.493 | 1.348 | 0.4257 |
| <50 | 38-63 K | 7.0-12.9% | Not Insured | Others vs. White | 1.679 | 0.673 | 4.187 | 0.2666 |
| <50 | 38-63 K | 7.0-12.9% | Private | Black vs. White | 1.409 | 1.249 | 1.59 | <.0001 |
| <50 | 38-63 K | 7.0-12.9% | Private | Asian vs. White | 1.051 | 0.818 | 1.35 | 0.6954 |
| <50 | 38-63 K | 7.0-12.9% | Private | Hispanic vs. White | 0.806 | 0.636 | 1.021 | 0.0733 |
| <50 | 38-63 K | 7.0-12.9% | Private | Others vs. White | 1.881 | 1.266 | 2.794 | 0.0018 |
| <50 | 38-63 K | <7.0% | Government | Black vs. White | 1.814 | 1.247 | 2.639 | 0.0019 |
| <50 | 38-63 K | <7.0% | Government | Asian vs. White | 0.729 | 0.256 | 2.075 | 0.5533 |
| <50 | 38-63 K | <7.0% | Government | Hispanic vs. White | 0.593 | 0.183 | 1.914 | 0.3817 |
| <50 | 38-63 K | <7.0% | Government | Others vs. White | 1.074 | 0.422 | 2.729 | 0.8813 |
| <50 | 38-63 K | <7.0% | Not Insured | Black vs. White | 1.118 | 0.581 | 2.152 | 0.7386 |
| <50 | 38-63 K | <7.0% | Not Insured | Hispanic vs. White | 0.989 | 0.454 | 2.157 | 0.9787 |
| <50 | 38-63 K | <7.0% | Private | Black vs. White | 1.02 | 0.766 | 1.358 | 0.8939 |
| <50 | 38-63 K | <7.0% | Private | Asian vs. White | 0.865 | 0.563 | 1.33 | 0.5092 |
| <50 | 38-63 K | <7.0% | Private | Hispanic vs. White | 0.685 | 0.407 | 1.152 | 0.1534 |
| <50 | 38-63 K | <7.0% | Private | Others vs. White | 0.988 | 0.44 | 2.218 | 0.9773 |
| <50 | ≥63 K | ≥21.0% | Government | Black vs. White | 0.527 | 0.144 | 1.931 | 0.3338 |
| <50 | ≥63 K | ≥21.0% | Government | Asian vs. White | 3.068 | 0.7 | 13.455 | 0.1372 |
| <50 | ≥63 K | ≥21.0% | Government | Hispanic vs. White | 1.666 | 0.48 | 5.782 | 0.4212 |
| <50 | ≥63 K | ≥21.0% | Government | Others vs. White | 0.069 | 0.003 | 1.553 | 0.0924 |
| <50 | ≥63 K | ≥21.0% | Private | Black vs. White | 1.48 | 0.902 | 2.428 | 0.1207 |
| <50 | ≥63 K | ≥21.0% | Private | Asian vs. White | 1.443 | 0.798 | 2.61 | 0.2255 |
| <50 | ≥63 K | ≥21.0% | Private | Hispanic vs. White | 1.031 | 0.644 | 1.649 | 0.9001 |
| <50 | ≥63 K | 13.0-20.9% | Government | Black vs. White | 0.948 | 0.56 | 1.605 | 0.8418 |
| <50 | ≥63 K | 13.0-20.9% | Government | Asian vs. White | 0.875 | 0.46 | 1.666 | 0.6853 |
| <50 | ≥63 K | 13.0-20.9% | Government | Hispanic vs. White | 0.99 | 0.601 | 1.629 | 0.9674 |
| <50 | ≥63 K | 13.0-20.9% | Government | Others vs. White | 2.26 | 0.279 | 18.277 | 0.4447 |
| <50 | ≥63 K | 13.0-20.9% | Not Insured | Black vs. White | 1.626 | 0.675 | 3.919 | 0.2786 |
| <50 | ≥63 K | 13.0-20.9% | Not Insured | Asian vs. White | 1.074 | 0.422 | 2.733 | 0.8805 |
| <50 | ≥63 K | 13.0-20.9% | Not Insured | Hispanic vs. White | 0.759 | 0.339 | 1.702 | 0.5035 |
| <50 | ≥63 K | 13.0-20.9% | Not Insured | Others vs. White | 0.751 | 0.07 | 8.081 | 0.8129 |
| <50 | ≥63 K | 13.0-20.9% | Private | Black vs. White | 1.033 | 0.783 | 1.361 | 0.8204 |
| <50 | ≥63 K | 13.0-20.9% | Private | Asian vs. White | 0.937 | 0.675 | 1.299 | 0.6947 |
| <50 | ≥63 K | 13.0-20.9% | Private | Hispanic vs. White | 1.179 | 0.887 | 1.567 | 0.2581 |
| <50 | ≥63 K | 13.0-20.9% | Private | Others vs. White | 1.236 | 0.504 | 3.032 | 0.643 |
| <50 | ≥63 K | 7.0-12.9% | Government | Black vs. White | 1.013 | 0.751 | 1.366 | 0.9322 |
| <50 | ≥63 K | 7.0-12.9% | Government | Asian vs. White | 1.007 | 0.703 | 1.442 | 0.9696 |
| <50 | ≥63 K | 7.0-12.9% | Government | Hispanic vs. White | 0.738 | 0.492 | 1.107 | 0.142 |
| <50 | ≥63 K | 7.0-12.9% | Government | Others vs. White | 1.637 | 0.729 | 3.673 | 0.2322 |
| <50 | ≥63 K | 7.0-12.9% | Not Insured | Black vs. White | 1.024 | 0.656 | 1.599 | 0.9166 |
| <50 | ≥63 K | 7.0-12.9% | Not Insured | Asian vs. White | 1.484 | 0.765 | 2.879 | 0.2428 |
| <50 | ≥63 K | 7.0-12.9% | Not Insured | Hispanic vs. White | 1.157 | 0.724 | 1.85 | 0.5414 |
| <50 | ≥63 K | 7.0-12.9% | Not Insured | Others vs. White | 1.499 | 0.328 | 6.864 | 0.6017 |
| <50 | ≥63 K | 7.0-12.9% | Private | Black vs. White | 1.384 | 1.195 | 1.602 | <.0001 |
| <50 | ≥63 K | 7.0-12.9% | Private | Asian vs. White | 1.006 | 0.84 | 1.204 | 0.9519 |
| <50 | ≥63 K | 7.0-12.9% | Private | Hispanic vs. White | 0.885 | 0.718 | 1.091 | 0.2535 |
| <50 | ≥63 K | 7.0-12.9% | Private | Others vs. White | 1.272 | 0.775 | 2.086 | 0.3416 |
| <50 | ≥63 K | <7.0% | Government | Black vs. White | 1.393 | 1.063 | 1.826 | 0.0163 |
| <50 | ≥63 K | <7.0% | Government | Asian vs. White | 0.733 | 0.472 | 1.138 | 0.1662 |
| <50 | ≥63 K | <7.0% | Government | Hispanic vs. White | 0.49 | 0.306 | 0.785 | 0.003 |
| <50 | ≥63 K | <7.0% | Government | Others vs. White | 1.323 | 0.646 | 2.71 | 0.4448 |
| <50 | ≥63 K | <7.0% | Not Insured | Black vs. White | 0.981 | 0.614 | 1.567 | 0.9356 |
| <50 | ≥63 K | <7.0% | Not Insured | Asian vs. White | 0.816 | 0.45 | 1.479 | 0.5023 |
| <50 | ≥63 K | <7.0% | Not Insured | Hispanic vs. White | 0.752 | 0.448 | 1.263 | 0.2818 |
| <50 | ≥63 K | <7.0% | Not Insured | Others vs. White | 0.485 | 0.148 | 1.591 | 0.2326 |
| <50 | ≥63 K | <7.0% | Private | Black vs. White | 1.157 | 1 | 1.34 | 0.0507 |
| <50 | ≥63 K | <7.0% | Private | Asian vs. White | 0.966 | 0.839 | 1.113 | 0.6352 |
| <50 | ≥63 K | <7.0% | Private | Hispanic vs. White | 0.856 | 0.688 | 1.064 | 0.1615 |
| <50 | ≥63 K | <7.0% | Private | Others vs. White | 0.645 | 0.41 | 1.015 | 0.0581 |
| ≥50 | <38 K | ≥21.0% | Government | Black vs. White | 0.958 | 0.933 | 0.985 | 0.0021 |
| ≥50 | <38 K | ≥21.0% | Government | Asian vs. White | 0.803 | 0.719 | 0.897 | 0.0001 |
| ≥50 | <38 K | ≥21.0% | Government | Hispanic vs. White | 0.713 | 0.681 | 0.747 | <.0001 |
| ≥50 | <38 K | ≥21.0% | Government | Others vs. White | 0.916 | 0.812 | 1.032 | 0.1498 |
| ≥50 | <38 K | ≥21.0% | Not Insured | Black vs. White | 0.939 | 0.842 | 1.047 | 0.259 |
| ≥50 | <38 K | ≥21.0% | Not Insured | Asian vs. White | 0.675 | 0.46 | 0.992 | 0.0452 |
| ≥50 | <38 K | ≥21.0% | Not Insured | Hispanic vs. White | 0.626 | 0.537 | 0.728 | <.0001 |
| ≥50 | <38 K | ≥21.0% | Not Insured | Others vs. White | 0.563 | 0.317 | 1 | 0.0499 |
| ≥50 | <38 K | ≥21.0% | Private | Black vs. White | 1.036 | 0.983 | 1.091 | 0.1854 |
| ≥50 | <38 K | ≥21.0% | Private | Asian vs. White | 0.841 | 0.674 | 1.049 | 0.1243 |
| ≥50 | <38 K | ≥21.0% | Private | Hispanic vs. White | 0.803 | 0.735 | 0.877 | <.0001 |
| ≥50 | <38 K | ≥21.0% | Private | Others vs. White | 1.082 | 0.846 | 1.383 | 0.5305 |
| ≥50 | <38 K | 13.0-20.9% | Government | Black vs. White | 0.964 | 0.926 | 1.005 | 0.0817 |
| ≥50 | <38 K | 13.0-20.9% | Government | Asian vs. White | 0.957 | 0.737 | 1.242 | 0.7417 |
| ≥50 | <38 K | 13.0-20.9% | Government | Hispanic vs. White | 0.76 | 0.664 | 0.871 | <.0001 |
| ≥50 | <38 K | 13.0-20.9% | Government | Others vs. White | 0.965 | 0.807 | 1.154 | 0.6947 |
| ≥50 | <38 K | 13.0-20.9% | Not Insured | Black vs. White | 0.973 | 0.81 | 1.168 | 0.7664 |
| ≥50 | <38 K | 13.0-20.9% | Not Insured | Asian vs. White | 0.692 | 0.281 | 1.706 | 0.424 |
| ≥50 | <38 K | 13.0-20.9% | Not Insured | Hispanic vs. White | 0.564 | 0.353 | 0.901 | 0.0166 |
| ≥50 | <38 K | 13.0-20.9% | Not Insured | Others vs. White | 1.41 | 0.516 | 3.85 | 0.5027 |
| ≥50 | <38 K | 13.0-20.9% | Private | Black vs. White | 1.014 | 0.939 | 1.094 | 0.7293 |
| ≥50 | <38 K | 13.0-20.9% | Private | Asian vs. White | 1.104 | 0.765 | 1.594 | 0.5972 |
| ≥50 | <38 K | 13.0-20.9% | Private | Hispanic vs. White | 0.885 | 0.677 | 1.158 | 0.3729 |
| ≥50 | <38 K | 13.0-20.9% | Private | Others vs. White | 1.053 | 0.685 | 1.619 | 0.8138 |
| ≥50 | <38 K | 7.0-12.9% | Government | Black vs. White | 0.997 | 0.884 | 1.125 | 0.963 |
| ≥50 | <38 K | 7.0-12.9% | Government | Asian vs. White | 0.733 | 0.46 | 1.169 | 0.1921 |
| ≥50 | <38 K | 7.0-12.9% | Government | Hispanic vs. White | 0.853 | 0.619 | 1.177 | 0.3329 |
| ≥50 | <38 K | 7.0-12.9% | Government | Others vs. White | 1.149 | 0.777 | 1.698 | 0.4866 |
| ≥50 | <38 K | 7.0-12.9% | Not Insured | Black vs. White | 0.444 | 0.191 | 1.034 | 0.0599 |
| ≥50 | <38 K | 7.0-12.9% | Not Insured | Asian vs. White | 0.593 | 0.119 | 2.962 | 0.5238 |
| ≥50 | <38 K | 7.0-12.9% | Not Insured | Hispanic vs. White | 0.609 | 0.196 | 1.891 | 0.3906 |
| ≥50 | <38 K | 7.0-12.9% | Not Insured | Others vs. White | 0.82 | 0.151 | 4.446 | 0.8183 |
| ≥50 | <38 K | 7.0-12.9% | Private | Black vs. White | 1.009 | 0.808 | 1.26 | 0.9395 |
| ≥50 | <38 K | 7.0-12.9% | Private | Asian vs. White | 1.347 | 0.635 | 2.856 | 0.4376 |
| ≥50 | <38 K | 7.0-12.9% | Private | Hispanic vs. White | 0.816 | 0.469 | 1.418 | 0.4702 |
| ≥50 | <38 K | 7.0-12.9% | Private | Others vs. White | 1.075 | 0.534 | 2.165 | 0.8399 |
| ≥50 | <38 K | <7.0% | Government | Black vs. White | 1.257 | 0.802 | 1.97 | 0.3179 |
| ≥50 | <38 K | <7.0% | Government | Asian vs. White | 0.748 | 0.38 | 1.471 | 0.4003 |
| ≥50 | <38 K | <7.0% | Government | Hispanic vs. White | 1.845 | 1.004 | 3.39 | 0.0484 |
| ≥50 | <38 K | <7.0% | Government | Others vs. White | 1.212 | 0.428 | 3.428 | 0.7172 |
| ≥50 | <38 K | <7.0% | Not Insured | Black vs. White | 0.963 | 0.022 | 41.546 | 0.9842 |
| ≥50 | <38 K | <7.0% | Private | Black vs. White | 1.719 | 0.857 | 3.445 | 0.127 |
| ≥50 | <38 K | <7.0% | Private | Asian vs. White | 0.271 | 0.036 | 2.017 | 0.2021 |
| ≥50 | <38 K | <7.0% | Private | Hispanic vs. White | 0.616 | 0.078 | 4.874 | 0.6458 |
| ≥50 | 38-63 K | ≥21.0% | Government | Black vs. White | 0.963 | 0.922 | 1.006 | 0.0874 |
| ≥50 | 38-63 K | ≥21.0% | Government | Asian vs. White | 0.75 | 0.698 | 0.805 | <.0001 |
| ≥50 | 38-63 K | ≥21.0% | Government | Hispanic vs. White | 0.77 | 0.735 | 0.807 | <.0001 |
| ≥50 | 38-63 K | ≥21.0% | Government | Others vs. White | 0.74 | 0.608 | 0.901 | 0.0027 |
| ≥50 | 38-63 K | ≥21.0% | Not Insured | Black vs. White | 1.128 | 0.959 | 1.328 | 0.1457 |
| ≥50 | 38-63 K | ≥21.0% | Not Insured | Asian vs. White | 0.851 | 0.643 | 1.126 | 0.2595 |
| ≥50 | 38-63 K | ≥21.0% | Not Insured | Hispanic vs. White | 0.725 | 0.616 | 0.852 | <.0001 |
| ≥50 | 38-63 K | ≥21.0% | Not Insured | Others vs. White | 0.882 | 0.452 | 1.723 | 0.7139 |
| ≥50 | 38-63 K | ≥21.0% | Private | Black vs. White | 0.967 | 0.898 | 1.041 | 0.3744 |
| ≥50 | 38-63 K | ≥21.0% | Private | Asian vs. White | 0.923 | 0.822 | 1.036 | 0.1729 |
| ≥50 | 38-63 K | ≥21.0% | Private | Hispanic vs. White | 0.88 | 0.816 | 0.949 | 0.0009 |
| ≥50 | 38-63 K | ≥21.0% | Private | Others vs. White | 0.795 | 0.596 | 1.06 | 0.1178 |
| ≥50 | 38-63 K | 13.0-20.9% | Government | Black vs. White | 0.967 | 0.936 | 1 | 0.048 |
| ≥50 | 38-63 K | 13.0-20.9% | Government | Asian vs. White | 0.769 | 0.699 | 0.846 | <.0001 |
| ≥50 | 38-63 K | 13.0-20.9% | Government | Hispanic vs. White | 0.767 | 0.722 | 0.815 | <.0001 |
| ≥50 | 38-63 K | 13.0-20.9% | Government | Others vs. White | 0.755 | 0.653 | 0.874 | 0.0002 |
| ≥50 | 38-63 K | 13.0-20.9% | Not Insured | Black vs. White | 0.945 | 0.828 | 1.078 | 0.398 |
| ≥50 | 38-63 K | 13.0-20.9% | Not Insured | Asian vs. White | 0.944 | 0.69 | 1.293 | 0.721 |
| ≥50 | 38-63 K | 13.0-20.9% | Not Insured | Hispanic vs. White | 0.773 | 0.627 | 0.954 | 0.0164 |
| ≥50 | 38-63 K | 13.0-20.9% | Not Insured | Others vs. White | 0.616 | 0.329 | 1.152 | 0.1295 |
| ≥50 | 38-63 K | 13.0-20.9% | Private | Black vs. White | 1.049 | 0.994 | 1.106 | 0.0833 |
| ≥50 | 38-63 K | 13.0-20.9% | Private | Asian vs. White | 0.781 | 0.675 | 0.904 | 0.0009 |
| ≥50 | 38-63 K | 13.0-20.9% | Private | Hispanic vs. White | 0.897 | 0.813 | 0.989 | 0.0293 |
| ≥50 | 38-63 K | 13.0-20.9% | Private | Others vs. White | 0.802 | 0.647 | 0.995 | 0.0446 |
| ≥50 | 38-63 K | 7.0-12.9% | Government | Black vs. White | 1.009 | 0.969 | 1.051 | 0.6651 |
| ≥50 | 38-63 K | 7.0-12.9% | Government | Asian vs. White | 0.705 | 0.637 | 0.782 | <.0001 |
| ≥50 | 38-63 K | 7.0-12.9% | Government | Hispanic vs. White | 0.801 | 0.74 | 0.867 | <.0001 |
| ≥50 | 38-63 K | 7.0-12.9% | Government | Others vs. White | 0.935 | 0.814 | 1.074 | 0.3389 |
| ≥50 | 38-63 K | 7.0-12.9% | Not Insured | Black vs. White | 0.868 | 0.728 | 1.036 | 0.1161 |
| ≥50 | 38-63 K | 7.0-12.9% | Not Insured | Asian vs. White | 0.875 | 0.584 | 1.311 | 0.517 |
| ≥50 | 38-63 K | 7.0-12.9% | Not Insured | Hispanic vs. White | 0.448 | 0.325 | 0.618 | <.0001 |
| ≥50 | 38-63 K | 7.0-12.9% | Not Insured | Others vs. White | 0.519 | 0.258 | 1.045 | 0.0664 |
| ≥50 | 38-63 K | 7.0-12.9% | Private | Black vs. White | 1.043 | 0.975 | 1.115 | 0.219 |
| ≥50 | 38-63 K | 7.0-12.9% | Private | Asian vs. White | 0.779 | 0.666 | 0.911 | 0.0018 |
| ≥50 | 38-63 K | 7.0-12.9% | Private | Hispanic vs. White | 0.865 | 0.756 | 0.989 | 0.0339 |
| ≥50 | 38-63 K | 7.0-12.9% | Private | Others vs. White | 0.856 | 0.648 | 1.13 | 0.2715 |
| ≥50 | 38-63 K | <7.0% | Government | Black vs. White | 1.013 | 0.923 | 1.111 | 0.7926 |
| ≥50 | 38-63 K | <7.0% | Government | Asian vs. White | 0.75 | 0.632 | 0.89 | 0.001 |
| ≥50 | 38-63 K | <7.0% | Government | Hispanic vs. White | 0.746 | 0.634 | 0.878 | 0.0004 |
| ≥50 | 38-63 K | <7.0% | Government | Others vs. White | 0.919 | 0.724 | 1.166 | 0.487 |
| ≥50 | 38-63 K | <7.0% | Not Insured | Black vs. White | 0.697 | 0.458 | 1.059 | 0.0909 |
| ≥50 | 38-63 K | <7.0% | Not Insured | Asian vs. White | 0.717 | 0.33 | 1.558 | 0.4007 |
| ≥50 | 38-63 K | <7.0% | Not Insured | Hispanic vs. White | 0.788 | 0.412 | 1.51 | 0.4734 |
| ≥50 | 38-63 K | <7.0% | Not Insured | Others vs. White | 0.393 | 0.11 | 1.411 | 0.1521 |
| ≥50 | 38-63 K | <7.0% | Private | Black vs. White | 1.044 | 0.9 | 1.209 | 0.5714 |
| ≥50 | 38-63 K | <7.0% | Private | Asian vs. White | 1.213 | 0.956 | 1.538 | 0.1115 |
| ≥50 | 38-63 K | <7.0% | Private | Hispanic vs. White | 0.956 | 0.737 | 1.24 | 0.7325 |
| ≥50 | 38-63 K | <7.0% | Private | Others vs. White | 0.883 | 0.58 | 1.344 | 0.5612 |
| ≥50 | ≥63 K | ≥21.0% | Government | Black vs. White | 0.912 | 0.758 | 1.096 | 0.3247 |
| ≥50 | ≥63 K | ≥21.0% | Government | Asian vs. White | 0.798 | 0.667 | 0.955 | 0.0136 |
| ≥50 | ≥63 K | ≥21.0% | Government | Hispanic vs. White | 0.684 | 0.574 | 0.815 | <.0001 |
| ≥50 | ≥63 K | ≥21.0% | Government | Others vs. White | 0.302 | 0.113 | 0.812 | 0.0176 |
| ≥50 | ≥63 K | ≥21.0% | Not Insured | Black vs. White | 0.658 | 0.218 | 1.989 | 0.4584 |
| ≥50 | ≥63 K | ≥21.0% | Not Insured | Asian vs. White | 0.596 | 0.233 | 1.527 | 0.2814 |
| ≥50 | ≥63 K | ≥21.0% | Not Insured | Hispanic vs. White | 0.475 | 0.187 | 1.205 | 0.117 |
| ≥50 | ≥63 K | ≥21.0% | Private | Black vs. White | 1.092 | 0.827 | 1.442 | 0.5357 |
| ≥50 | ≥63 K | ≥21.0% | Private | Asian vs. White | 0.819 | 0.621 | 1.081 | 0.1586 |
| ≥50 | ≥63 K | ≥21.0% | Private | Hispanic vs. White | 0.801 | 0.63 | 1.018 | 0.0702 |
| ≥50 | ≥63 K | ≥21.0% | Private | Others vs. White | 1.431 | 0.586 | 3.495 | 0.4314 |
| ≥50 | ≥63 K | 13.0-20.9% | Government | Black vs. White | 0.87 | 0.786 | 0.962 | 0.0069 |
| ≥50 | ≥63 K | 13.0-20.9% | Government | Asian vs. White | 0.78 | 0.696 | 0.874 | <.0001 |
| ≥50 | ≥63 K | 13.0-20.9% | Government | Hispanic vs. White | 0.696 | 0.616 | 0.788 | <.0001 |
| ≥50 | ≥63 K | 13.0-20.9% | Government | Others vs. White | 0.796 | 0.581 | 1.092 | 0.158 |
| ≥50 | ≥63 K | 13.0-20.9% | Not Insured | Black vs. White | 1.198 | 0.68 | 2.11 | 0.5319 |
| ≥50 | ≥63 K | 13.0-20.9% | Not Insured | Asian vs. White | 0.885 | 0.485 | 1.615 | 0.6899 |
| ≥50 | ≥63 K | 13.0-20.9% | Not Insured | Hispanic vs. White | 1.004 | 0.599 | 1.68 | 0.9893 |
| ≥50 | ≥63 K | 13.0-20.9% | Not Insured | Others vs. White | 1.03 | 0.134 | 7.929 | 0.9771 |
| ≥50 | ≥63 K | 13.0-20.9% | Private | Black vs. White | 1.01 | 0.876 | 1.164 | 0.8937 |
| ≥50 | ≥63 K | 13.0-20.9% | Private | Asian vs. White | 0.896 | 0.764 | 1.051 | 0.1787 |
| ≥50 | ≥63 K | 13.0-20.9% | Private | Hispanic vs. White | 0.822 | 0.695 | 0.972 | 0.022 |
| ≥50 | ≥63 K | 13.0-20.9% | Private | Others vs. White | 0.833 | 0.492 | 1.412 | 0.4975 |
| ≥50 | ≥63 K | 7.0-12.9% | Government | Black vs. White | 0.984 | 0.927 | 1.044 | 0.5865 |
| ≥50 | ≥63 K | 7.0-12.9% | Government | Asian vs. White | 0.792 | 0.736 | 0.853 | <.0001 |
| ≥50 | ≥63 K | 7.0-12.9% | Government | Hispanic vs. White | 0.792 | 0.729 | 0.86 | <.0001 |
| ≥50 | ≥63 K | 7.0-12.9% | Government | Others vs. White | 0.762 | 0.605 | 0.958 | 0.0203 |
| ≥50 | ≥63 K | 7.0-12.9% | Not Insured | Black vs. White | 0.96 | 0.748 | 1.233 | 0.7489 |
| ≥50 | ≥63 K | 7.0-12.9% | Not Insured | Asian vs. White | 0.518 | 0.351 | 0.765 | 0.0009 |
| ≥50 | ≥63 K | 7.0-12.9% | Not Insured | Hispanic vs. White | 0.77 | 0.568 | 1.045 | 0.094 |
| ≥50 | ≥63 K | 7.0-12.9% | Not Insured | Others vs. White | 0.927 | 0.378 | 2.273 | 0.8685 |
| ≥50 | ≥63 K | 7.0-12.9% | Private | Black vs. White | 1.054 | 0.968 | 1.148 | 0.2254 |
| ≥50 | ≥63 K | 7.0-12.9% | Private | Asian vs. White | 0.933 | 0.842 | 1.034 | 0.1848 |
| ≥50 | ≥63 K | 7.0-12.9% | Private | Hispanic vs. White | 0.958 | 0.85 | 1.08 | 0.4862 |
| ≥50 | ≥63 K | 7.0-12.9% | Private | Others vs. White | 0.628 | 0.454 | 0.869 | 0.005 |
| ≥50 | ≥63 K | <7.0% | Government | Black vs. White | 0.958 | 0.904 | 1.014 | 0.141 |
| ≥50 | ≥63 K | <7.0% | Government | Asian vs. White | 0.814 | 0.76 | 0.873 | <.0001 |
| ≥50 | ≥63 K | <7.0% | Government | Hispanic vs. White | 0.829 | 0.76 | 0.903 | <.0001 |
| ≥50 | ≥63 K | <7.0% | Government | Others vs. White | 0.675 | 0.571 | 0.797 | <.0001 |
| ≥50 | ≥63 K | <7.0% | Not Insured | Black vs. White | 0.963 | 0.743 | 1.248 | 0.7761 |
| ≥50 | ≥63 K | <7.0% | Not Insured | Asian vs. White | 0.8 | 0.589 | 1.088 | 0.1548 |
| ≥50 | ≥63 K | <7.0% | Not Insured | Hispanic vs. White | 0.814 | 0.58 | 1.142 | 0.233 |
| ≥50 | ≥63 K | <7.0% | Not Insured | Others vs. White | 0.876 | 0.478 | 1.606 | 0.6684 |
| ≥50 | ≥63 K | <7.0% | Private | Black vs. White | 1.052 | 0.964 | 1.147 | 0.2554 |
| ≥50 | ≥63 K | <7.0% | Private | Asian vs. White | 0.965 | 0.878 | 1.06 | 0.4574 |
| ≥50 | ≥63 K | <7.0% | Private | Hispanic vs. White | 0.978 | 0.861 | 1.111 | 0.737 |
| ≥50 | ≥63 K | <7.0% | Private | Others vs. White | 0.825 | 0.648 | 1.051 | 0.1194 |

**Supplemental Table 1.** Multivariable Cox models of outcomes by race in early-onset and late-onset CRC patients stratified by insurance status, community income and education level. Only p ≤ 0.0002 was considered statistically significant after Bonferroni correction. Abbreviations: HS=high school; OS=overall survival. LCL=lower confidence interval limit; UCL=upper confidence interval limit; K=1,000.
